# Supplementary material for: Device-Aided Therapies in Parkinson’s Disease—Results from the German Care4PD Study
Source: Brain Sci. 2023 Apr 28;13(5):736. doi: 10.3390/brainsci13050736 (PMC10216485; doi:10.3390/brainsci13050736)
Supplement: Supplementary file 1 [file brainsci-13-00736-s001.zip › brainsci-2293902-supplementary.pdf]

Supplementary Material

Care4PD - Questionnaire for people with Parkinson’s Disease

Dear ladies and gentlemen,
please fill out the following questionnaire evaluating the care situation in Parkinson's disease.

Participation

- **Every Parkinson's patient**
- Anonymous data collection, no indication of personal data
- Please take as much time as your need (if necessary, fill out the questionnaire stepwise)

Aims

- **Evaluate the care and supply situation in Parkinson's disease**
- Identify supply gaps
- Collect patient´s request

Return questionnaire

- **Until July 15, 2021**
- Please send the completed questionnaire in the enclosed envelope
- Please do not specify a sender

Instructions for completing the questionnaire

- Please read all questions carefully and fill them out legibly with a **dark** pen/ballpoint pen (no pencil).
- Please note: There are questions with only one possible answer and those with multiple answers.
- Please tick like this:

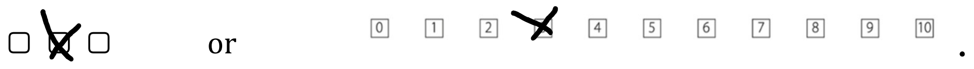

- Please make corrections like this:

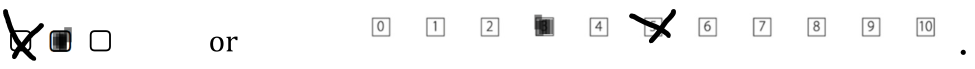

- Please enter numbers like this: 1 2 3
- For questions that do not apply to you, please tick "Not applicable".
- Please **do not** separate the sheets of the questionnaire (2 sheets of 4 pages each = 8 pages in total) in the perforation in the middle. Please send both forms back together.

Thank you very much for your participation.

Best regards,

Dr. med. Odette Fründt
Resident Physician
Research Parkinson's and Movement Disorders,
Department of Neurology

Prof. Dr. med. Martin Südmeyer
Chief Physician
Department of Neurology

Part 1: General questions – all participants

1. **Who filled out the questionnaire predominantly?**

▢ Patient ▢ Relatives/Family member ▢ Others ▢ Nursing staff

2. **Approximately how many residents does your current place of residence have?**

▢ Less than 5000 ▢ 5000 – 19 9999 ▢ 20 000 – 100 000 ▢ More than 100 000

3. **Your gender?** ▢ Male ▢ Female ▢ Diverse

4. **How old are you?** __________ Years

5. **Your age when you received the Parkinson's diagnosis?** __________ Years

6. **During the last 2 weeks: How impaired did you feel by your Parkinson's disease?**

Not at all Very much

[0] [1] [2] [3] [4] [5] [6] [7] [8] [9] [10]

7. **How would you describe your <u>current</u> PD stadium?** → Hoehn & Yahr stage

▢ Only one-sided symptoms

▢ Bilateral symptoms without imbalance

▢ Mild to moderate bilateral symptoms with mild imbalance, physically independent

▢ Severe impairment, able to walk and stand unaided

▢ I am mostly dependent on a wheelchair or bedridden

8. **Which PD symptoms bother you the most?** (Please tick a maximum of 3)

▢ Stiffness, reduced mobility, slowdown (brady- or akinesia)

▢ Gait and balance problems with frequent falls

▢ Gait and balance problems with infrequent or no falls

▢ Fluctuation between good and bad mobility

▢ Involuntary movements

▢ Autonomic dysfunction (e.g., constipation, drooling, incontinence, low blood pressure)

▢ Memory problems or other similar problems (e.g., orientation problems)

▢ Glumness, joylessness, lack of motivation or anxiety)

▢ Impulse control disorder (e.g., eating addiction, gambling addiction, shopping addiction, hypersexuality)

▢ Trembling / tremor

▢ Problems with speech or swallowing

▢ Sleep disorders

▢ Hallucinations

▢ Other symptoms (please name it): ____________________

9. **How many percentages of hours within the waking time do you typically have episodes with low level or OFF motor functioning?** → MDS-UPDRS III

▢ No Off time ▢ Less than 25% of waking hours ▢ 26% - 50% of waking hours

▢ 51% - 75% of waking hours ▢ More than 75% of waking hours ▢ I don´t know

10. Due to having PD, how often during the last month have you…? (Please tick one box for each question) → PDQ-8

	never	occasionally	sometimes	often	Always/ can`t do it at all
…had difficulty getting around in public	☐	☐	☐	☐	☐
…had difficulty dressing yourself	☐	☐	☐	☐	☐
…felt depressed	☐	☐	☐	☐	☐
…had problems with your close personal relationships?	☐	☐	☐	☐	☐
…had problems with your concentration, e.g., when reading or watching TV	☐	☐	☐	☐	☐
…felt unable to communicate with people properly?	☐	☐	☐	☐	☐
…had painful muscle cramps or spasms?	☐	☐	☐	☐	☐
…felt embarrassed in public due to having PD?	☐	☐	☐	☐	☐

11. Did you have to be admitted to a hospital due to your PD during the last 6 months?

☐ Yes, elective admission ☐ Yes, unplanned admission ☐ No

12. Who does support you primarily?

☐ Spouse or cohabitant ☐ Relatives ☐ Professional nursing staff ☐ Friends

☐ Colleagues ☐ No one ☐ Not applicable (don't need support)

13. How independent are you regarding the following activities of daily living? (Please tick one box for each activity) → Katz index

Bathing/showering	▢ Independence	▢ Dependence (with help)
Dressing	▢ Independence	▢ Dependence (with help)
Continence	▢ Independence	▢ Dependence (with help)
Toileting	▢ Independence	▢ Dependence (with help)
Transferring (e.g., out of bed or chair)	▢ Independence	▢ Dependence (with help)
Feeding	▢ Independence	▢ Dependence (with help)

14. Which PD therapy do you currently receive? (Multiple answers possible)

▢ PD medication (pills or patch)

▢ Speech and swallowing therapy

▢ Deep brain stimulation (DBS)

▢ Occupational therapy incl. optimizing ADL

▢ Apomorphine pump or pen

▢ I don´t have any of those professional therapies

▢ LCIG (Duodopa®) pump

▢ Others (e. g. psychotherapy, music therapy…)

▢ Physiotherapy

15. How often (per day) do you take your PD medication (pill/patch)?

▢ 1x/Day ▢ 2-3x/Day ▢ 4-6x/Day ▢ More than 6x/Day ▢ No medication

16. Who is mainly responsible for your PD medication?

▢ Myself ▢ Nursing staff ▢ Other (e. g. family members) ▢ Friends ▢ Not applicable

17. Do you take or receive your medication according to the medication regimen or your doctor´s time specification?

▢ Always ▢ Often ▢ Sometimes ▢ Occasionally ▢ Never

▢ Don´t know ▢ Not applicable (no medication)

18. Regarding your PD, do you feel bad or impaired when you didn´t take medication on time?

▢ Always ▢ Often ▢ Sometimes ▢ Occasionally ▢ Never

▢ Don´t know ▢ Not applicable (no medication)

19. Can you or your family members handle the DBS handheld device or pump?

Not at all Very good

0 1 2 3 4 5 6 7 8 9 10

▢ Not applicable (have no DBS or pump)

20. Can your nursing staff handle the DBS handheld device or pump?

Not at all Very good

0 1 2 3 4 5 6 7 8 9 10

▢ Not applicable (have no DBS or pump)

21. Does your general practitioner or neurologist offer domiciliary visits? (Please tick one box for each question)

	Yes	No	Don´t have one	Don´t know
General practitioner	▢	▢	▢	▢
Neurologist	▢	▢	▢	▢

22. How often do you visit (or stay in contact with) your general practitioner or neurologist? (Please tick one box for each question)

	Quarterly	Semi-annually	Annually	No contact	Don´t know
General practitioner	▢	▢	▢	▢	▢
Neurologist	▢	▢	▢	▢	▢

23. What kind of professional long-term care do you receive?

▢ Mobile nursing service at home ▢ Nursing home ▢ Self-organized care (e. g. 24-hour care)

▢ Not applicable

24. Do you have a care degree?

▢ None ▢ 1 ▢ 2 ▢ 3 ▢ 4 ▢ 5 ▢ Don´t know

25. At what age did you receive a care degree?

___________ Years ▢ Not applicable

26. Is the care degree sufficient to give you the support that you need?

▢ Yes ▢ No ▢ Not applicable

27. Do you know that there is PD specific nursing staff?

▢ Yes ▢ No

28. How important is it for you that your nursing staff…? (Please tick one box for each question)

	Not at all										Very important
…has PD specialist knowledge/skills	0	1	2	3	4	5	6	7	8	9	10

…proofs PD specialist knowledge/skills (e.g., with certificate) ☐0 ☐1 ☐2 ☐3 ☐4 ☐5 ☐6 ☐7 ☐8 ☐9 ☐10

29. Do you have any suggestions to improve care situation for you and other PD patients?

____ ▢ No suggestions

30. Did your PD deteriorate during the Corona pandemic?

Not at all — Very good

☐0 ☐1 ☐2 ☐3 ☐4 ☐5 ☐6 ☐7 ☐8 ☐9 ☐10

31. Have you been diagnosed with a COVID-19 infection?

▢ Yes, with symptoms ▢ Yes, without symptoms ▢ No

32. Would you get vaccinated against Corona?

▢ Yes, I am already vaccinated ▢ Yes, I wish to be vaccinated ▢ Maybe ▢ No

33. Overall, how concerned are you about the Corona pandemic?

Not at all — Very much

☐0 ☐1 ☐2 ☐3 ☐4 ☐5 ☐6 ☐7 ☐8 ☐9 ☐10

34. How much impact does the Corona pandemic have on your everyday life?

Not at all — Very often

☐0 ☐1 ☐2 ☐3 ☐4 ☐5 ☐6 ☐7 ☐8 ☐9 ☐10

35. Are there phases during the Corona Pandemic in which you have significantly less support in everyday life than average?

Not at all — Very strong

0 ☐ 1 ☐ 2 ☐ 3 ☐ 4 ☐ 5 ☐ 6 ☐ 7 ☐ 8 ☐ 9 ☐ 10 ☐

36. Compared with the time before the Corona pandemic: How much do you stay in contact with…? (Please tick one box for each question)

	Similarly frequent	Little less frequent	Significantly less frequent	Rare or no contact	Not applicable
…family members?	☐	☐	☐	☐	☐
…physicians?	☐	☐	☐	☐	☐
…therapists?	☐	☐	☐	☐	☐
…nursing staff?	☐	☐	☐	☐	☐
…other PD Patients?	☐	☐	☐	☐	☐

37. Which impairments do you feel during the Corona pandemic? (Multiple answers possible)

☐ No impairment

☐ Less contact with family members or others

☐ Less contact with physicians

☐ Less contact with therapists

☐ Less contact with nursing staff

☐ No or too few opportunities to leave house or room

☐ Other impairments (please name): ____

38. Can you use telemedicine during the Corona Pandemic? (e.g., telephone or web-based visits)?

Not at all — Very frequently

☐0 ☐1 ☐2 ☐3 ☐4 ☐5 ☐6 ☐7 ☐8 ☐9 ☐10

39. Would you like to use telemedicine (e.g., telephone or web-based visits) on a regular base?

☐ Yes, but only additionally to personal doctor's visit. ☐ Yes, as an alternative to personal doctor's visit. ☐ No.

40. Do you have the technical options to perform telemedicine (e.g., telephone or web-based visits)?

☐ Yes, telephone & internet ☐ Yes, telephone only ☐ Yes, internet only

☐ No.

Part 2: Specific questions – p-LTC group only

41. Since when do you make use of professional care (e.g., mobile nursing service, nursing home, professional 24-hour care)?

Since___________ Years ☐ Not applicable

42. What is your share of the care costs?

☐ No additional costs ☐ 1000 to 2000 €

☐ Less than 500 € ☐ More than 2000 €

☐ 500 to 999 € ☐ Don´t know

43. How is your care treatment organized?

☐ Same staff and permanent contact person

☐ Occasionally changing staff, but permanent contact person

☐ Frequently changing staff, no permanent contact person

44. How often does the nursing care staff visit you?

☐ Once per week (or less) ☐ 2 to 3 times per day

☐ Several times per week ☐ More than 3 times per day

☐ Once per day

45. How much time does the nursing staff spent with you each day <u>on average</u>?

___________ minutes per day

46. Is the frequency and time that the nursing staff spends with you adequate/enough?

☐ Always ☐ Often ☐ Sometimes ☐ Occasionally ☐ Never

47. Regarding your nursing staff: Do you get the help that you need?

☐ Always ☐ Often ☐ Sometimes ☐ Occasionally ☐ Never

48. Are you frequently treated by nursing staff with an immigration background?

Yes (please name mother tongue)______________________________ ☐ No/not applicable

49. Are there any language barriers between you and your nursing staff?

☐ Yes ☐ No ☐ Partly ☐ Not applicable

50. How do you rate the knowledge of your nursing staff regarding…? (Please tick one box for each question)

	None										Very good
…PD in general?	0	1	2	3	4	5	6	7	8	9	10
… the PD therapy?	0	1	2	3	4	5	6	7	8	9	10

51. Did you choose your professional care due to their PD specific knowledge?

☐ Yes ☐ Yes, but no such institution found

▢ Yes, but other factors were more important ▢ No

52. Does your nursing staff participate in PD specific trainings?

▢ Yes ▢ No ▢ Don´t know

53. Does your nursing staff / institution procure adjunctive therapies?

▢ Yes, therapists belong to the nursing institution (internal)

▢ Yes, therapists do not belong to the nursing institution (external)

▢ Yes, internal and external therapists

▢ No

▢ Don´t know/not applicable

54. How well do you feel <u>protected</u> by the precautions given by your nursing staff against Corona virus?

Not at all Very well

0	1	2	3	4	5	6	7	8	9	10

55. How well do you feel <u>informed</u> about the precautions given by your nursing staff against Corona virus?

Not at all Very well

0	1	2	3	4	5	6	7	8	9	10

56. Which sanitary measures are used during your professional care sessions? (Multiple answers possible)

▢ Keep distance

▢ Hand hygiene (washing and disinfection of hands on a regular base)

▢ General hygiene measures (e.g., coughing / sneezing in the crook of the arm)

▢ Nursing staff wears a mask during care sessions

▢ Me, the patient, wears a mark during care sessions

▢ My family/relatives wear masks when come to visit me

▢ Limitation/interdiction of visitations

▢ Room ventilation

▢ Testing for COVID-19 in patients and/or nursing staff (e.g., PCR, quick tests)

▢ Don’ t know

▢ Others, please specify: ……………………………………………………………………
